# Sustained Delivery of Insulin-Like Growth Factor-1/Hepatocyte Growth Factor Stimulates Endogenous Cardiac Repair in the Chronic Infarcted Pig Heart

**DOI:** 10.1007/s12265-013-9518-4

**Published:** 2014-01-07

**Authors:** Stefan Koudstaal, Maartje M. C. Bastings, Dries A. M. Feyen, Cheryl D. Waring, Frebus J. van Slochteren, Patricia Y. W. Dankers, Daniele Torella, Joost P. G. Sluijter, Bernardo Nadal-Ginard, Pieter A. Doevendans, Georgina M. Ellison, Steven A. J. Chamuleau

**Affiliations:** 1 Department of Cardiology, Division Heart and Lungs, University Medical Center Utrecht, Room E03.511, PO Box 85500, 3508 GA Utrecht, The Netherlands; 2 Interuniversity Cardiology Institute of the Netherlands (ICIN), Utrecht, The Netherlands; 3 Biological Chemistry & Molecular Pharmacology, Dana-Farber Cancer Institute, Harvard, Boston, MA USA; 4 Institute for Complex Molecular Systems, Eindhoven University of Technology, Eindhoven, The Netherlands; 5 Laboratory of Chemical Biology, Eindhoven University of Technology, Eindhoven, The Netherlands; 6 The Stem Cell and Regenerative Biology Unit (BioStem), Liverpool JM University, Liverpool, UK; 7 Molecular and Cellular Cardiology, Department of Medicine, Magna Graecia University, Catanzaro, Italy; 8 Centre of Human and Aerospace Physiological Sciences & Centre for Stem Cells and Regenerative Medicine, School of Biomedical Sciences, King’s College, London, Guy’s Campus, London, SE1 1UL UK

**Keywords:** Cardiac stem/progenitor cells, IGF-1, HGF, Chronic myocardial infarction, Regeneration

## Abstract

Activation of endogenous cardiac stem/progenitor cells (eCSCs) can improve cardiac repair after acute myocardial infarction. We studied whether the in situ activation of eCSCs by insulin-like growth factor 1 (IGF-1) and hepatocyte growth factor (HGF) could be increased using a newly developed hydrogel in chronic myocardial infarction (MI). One-month post-MI pigs underwent NOGA-guided intramyocardial injections of IGF-1/HGF (GF: both 0.5 μg/mL, *n* = 5) or IGF-1/HGF incorporated in UPy hydrogel (UPy-GF; both 0.5 μg/mL, *n* = 5). UPy hydrogel without added growth factors was administered to four control (CTRL) pigs. Left ventricular ejection fraction was increased in the UPy-GF and GF animals compared to CTRLs. UPy-GF delivery reduced pathological hypertrophy, led to the formation of new, small cardiomyocytes, and increased capillarization. The eCSC population was increased almost fourfold in the border zone of the UPy-GF-treated hearts compared to CTRL hearts. These results show that IGF-1/HGF therapy led to an improved cardiac function in chronic MI and that effect size could be further increased by using UPy hydrogel.

**Electronic supplementary material:**

The online version of this article (doi:10.1007/s12265-013-9518-4) contains supplementary material, which is available to authorized users.

## Introduction

Despite early revascularization, acute myocardial infarction leads to irreversible loss of cardiomyocytes. As a consequence, the increased workload on the surviving cardiomyocytes often initiates a cascade of additional cardiomyocyte loss, myocardial remodeling, until the vicious circle ends in chronic heart failure (CHF) [1]. In the USA alone, approximately 5.7 million patients have CHF accounting for roughly US $30 billion annually in health care costs in 2008, which are predicted to triplicate by 2030 [2]. Given the initial loss of functional cardiomyocytes as the trigger of adverse remodeling processes that eventually lead to CHF [1], it is imperative to develop new low-cost, widely available treatments that are able to ameliorate the natural disease progression following a myocardial infarction (MI) to reduce the occurrence of post-MI heart failure.

One of the emerging therapeutic approaches relies on the notion that the adult mammalian heart fosters an innate capacity for cardiomyocyte regeneration, and different approaches to upscale this phenomenon to a clinically relevant level of myocardial regeneration are under intense investigation [3, 4].

The presence of tissue-specific, endogenous cardiac stem/progenitor cells (eCSCs) that reside in the heart and, upon activation, can create progeny that mature into functional cardiomyocytes and vasculature has been put forward as the causal agent for the regenerative capacity of the heart [5–7]. Recently, accumulating evidence supported the notion that the regenerative response of eCSCs toward the ischemic myocardium can be stimulated by means of in situ administration of various growth factors, such as insulin-like growth factor 1 (IGF-1) and hepatocyte growth factor (HGF) [8–10]. We have previously shown that the co-administration of IGF-1 and HGF led to the activation of eCSCs, increased cardiomyogenesis, and significantly improved cardiac function [9]. Yet, like previous studies, these results were reported on animal models addressing the acute phase of the MI, which, by itself, is a complex and powerful initiator of numerous molecular signaling processes in response to the ischemic insult [11]. Given the unmet clinical need for the development of new therapeutics to treat post-MI heart failure, we investigated whether the effect of IGF-1/HGF therapy is also effective in the post-MI heart, in which cardiac adverse remodeling is already an active process. To this end, we used the pig model of chronic MI as the pig heart closely resembles the human size and hemodynamics.

Besides the validation and identification of growth factors and signaling pathways that can stimulate cardiac repair, novel drug delivery systems such as biomaterials are extensively being studied to increase effect size. Previous reports showed that by combining growth factors with an injectable biomaterial, the biomaterial could serve as a controlled drug release platform, thereby improving functional outcome [10, 12]. Therefore, we investigated the added value of incorporating the growth factors within a smart hydrogel that can serve as a release scaffold upon catheter-based delivery in the infarcted heart to generate sustained GF levels at the site of dysfunction over time. Recently, we have reported on a new pH-switchable and self-healing hydrogel carrier that could be injected in the heart by transendocardial delivery using the NOGA™ catheter system (Biosense Webster, Johnson & Johnson Co.). We have previously shown that the release kinetics in vitro showed a 4-day time span for both IGF-1 and HGF in the absence of protein degradation based on the increased pH of the hydrogel [13]. Furthermore, IGF-1/HGF-loaded hydrogel injections in the border zone of the infarct created an effective spatial gradient of growth factors within the heart in which growth factor concentrations increased toward the site of injection [13].

Here, we present the first results on the efficacy of this new hydrogel system in combination with growth factors IGF-1/HGF on cardiac function and the progression of post-MI adverse remodeling in the pig chronic MI model.

## Methods

A detailed method section can be found in the Electronic supplementary material (ESM). Briefly, MI was induced by 75-min intracoronary balloon occlusion of the left circumflex (LCx) followed by reperfusion in 6-month-old female Dalland landrace pigs (∼70 kg). Four weeks later, ten intramyocardial injections of 0.2 mL each were placed in the infarct border zone with either IGF-1/HGF in 0.9 % saline (GF: both 0.5 μg/mL), IGF-1/HGF in UPy hydrogel (UPy-GF; both 0.5 μg/mL), or UPy hydrogel alone as a control (CTRL). Four weeks after treatment, cardiac function was assessed with 2D and 3D echocardiography and pressure volume loop analysis. Regional microvascular resistance was quantified by simultaneous assessment of the intracoronary pressure/and flow velocity parameters. Cardiomyocyte hypertrophy, cell proliferation, new cardiomyocyte and capillary formation, c-kit^pos^ CD45^neg^ eCSC number, and their committed progeny were characterized by immunohistochemistry and confocal microscopy.

## Results

### Mortality and Procedural Data

**Fig. 1:**
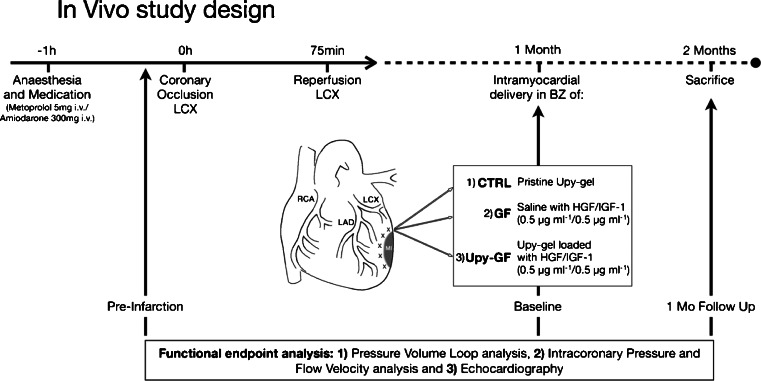
Study design. **a** Schematic study design showing the targeted intramyocardial delivery in the MI border zone of empty UPy-hydrogel as control (*1*, *CTRL*); IGF-1/HGF dissolved in saline, denoted as *GF* (*2*); or UPy hydrogel with IGF-1/HGF, denoted as *UPy-GF* (*3*)

A schematic of the study design is depicted in Fig. 1a. Three animals died during the induction of ischemia by LCx occlusion as a consequence of refractory ventricular fibrillation. One animal died 4 weeks later, prior to the intervention. Of the surviving 14 animals, five animals were randomly allocated to UPy-GF, five animals to GF, and the remaining four animals to UPy hydrogel alone, serving as controls.

### IGF-1/HGF Administration Improves Cardiac Function in Chronic MI

**Fig. 2:**
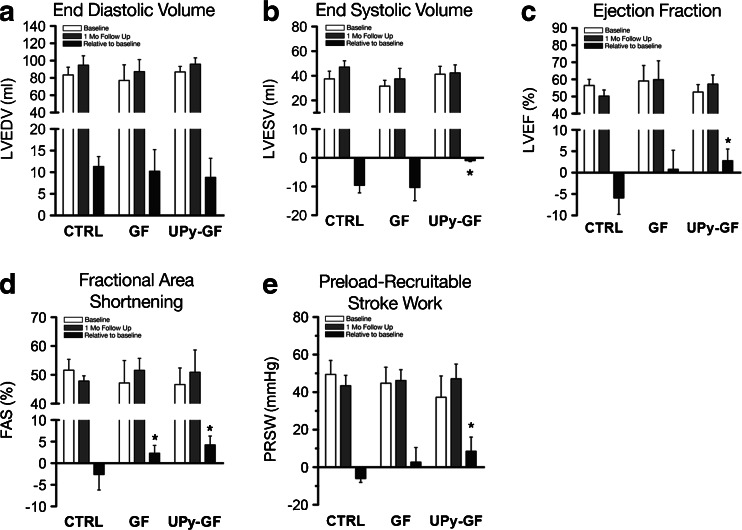
UPy-IGF-1/HGF therapy improves cardiac function in chronic MI. **a**, **b** LV end-diastolic and end-systolic volumes at baseline, 1 month after injection, and the relative change between both time points. **c** LV ejection fraction. **d** FAS measured by 2DE at the level of the papillary muscles. **e** Preload recruitable stroke work (*PRSW*) measured by intracardiac pressure–volume loop recordings. *p < 0.05 (vs. CTRL). All data are the mean ± SD; *n* = 3, 5, and 5 for CTRL, GF, and UPy-GF, respectively

First, the controls, UPy hydrogel without growth factors, were compared against a historical cohort of identical MI procedure and NOGA injections with phosphate-buffered saline 1 month after MI. There were no differences in any echocardiographic or PV loop-derived parameters (ESM Fig. 1). Thus, with no indication that the UPy hydrogel by itself influenced post-MI remodeling, we considered the empty UPy hydrogel as negative controls. As a reference value, prior to MI, the left ventricular end-diastolic volume (LVEDV) was on average 81.2 ± 6 mL. Two months after MI, there was a slight increase in LVEDV by ∼15 % in all groups, but it did not differ between treatment groups (CTRL vs. GF vs. UPy-GF, 94.9 ± 10.8 vs. 94.0 ± 8.9 vs. 92.4 ± 6.6 mL, respectively, *p* = 0.915; Fig. 2a). On the other hand, the mean change relative to baseline in LV end-systolic volumes (ESV) was significantly improved in the UPy-GF group compared to GF-treated animals and controls (−1.1 ± 2.3 vs. −10.3 ± 9.9 vs. −9.5 ± 4.6 mL, *p* = 0.03; Fig. 2b). Likewise, progressive deterioration in left ventricular ejection fraction was also significantly reversed in the UPy-GF group (mean change, +2.8 ± 2.7 %; Fig. 2c) compared to CTRL animals (−5.9 ± 3.8 %, *p* = 0.02; Fig. 2d), but did not significantly differ from the GF group (0.8 ± 2.0 %, *p* = 0.410; Fig. 2c). Fractional area shortening (ESM Fig. 2) was significantly improved in both the GF and UPy-GF groups compared to the CTRL animals (+2.3 ± 1.8 vs. +4.2 ± 2.0 vs. −2.6 ± 3.6 %, *p* = 0.008; Fig. 2d). With regard to diastolic function of the heart, the ratio of transmitral flow velocity to annular peak diastolic velocity (*E*/*E*′) was preserved in the IGF-1/HGF-treated animals (GF, 7.7 ± 0.3; UPy-GF, 7.4 ± 1.1) compared to CTRLs (9.3 ± 0.6, *p* = 0.04).

### Targeted Intramyocardial IGF-1/HGF Delivery Attenuates Cardiomyocyte Hypertrophy and Fibrosis in Chronic MI

**Fig. 3:**
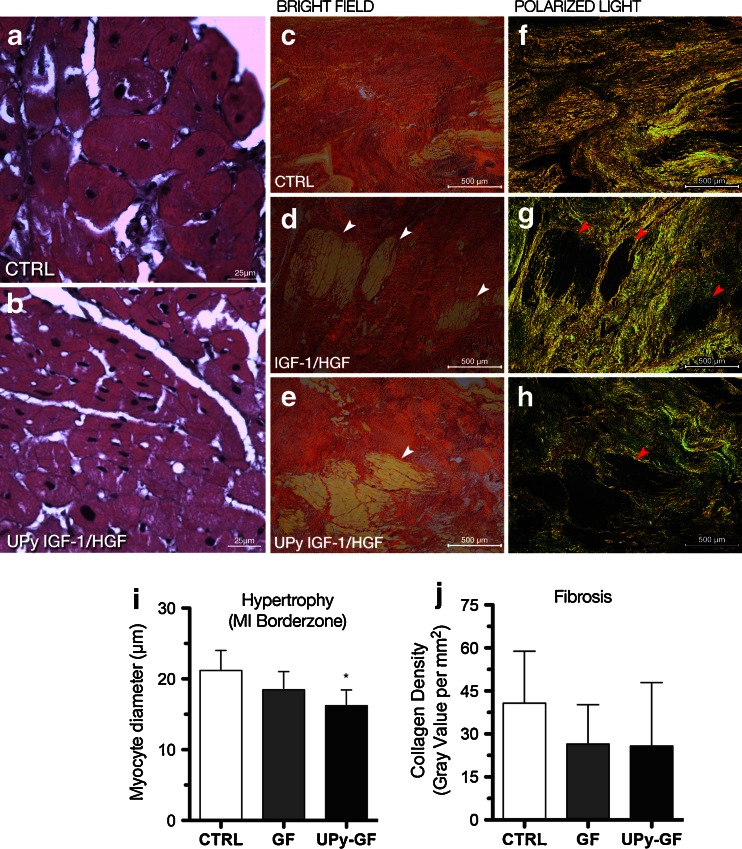
IGF-1/HGF treatment reduced pathological hypertrophy in the MI border zone. **a**, **b** Representative MI border zone sections (hematoxylin and eosin staining) showing adverse cardiac hypertrophy in the control-treated animals (**a**), which was not observed in the UPy-GF-treated animals (**b**). **c**–**h** Picric Sirius red staining in bright-field images (**c**–**e**) and under polarized light (**f**–**h**) showing extensive scar tissue in all groups depicted as *red staining* in bright-field microscopy. Under polarized light, color depended on the collagen fiber density (*yellow* for higher intensity, *green* for lower intensity). In both growth factor-treated groups, small myocardial islands were visible in the infarct area (see *arrowheads*). Quantification of cardiomyocyte diameter in the MI border zone (**i**) and fibrosis (**j**). *p < 0.05 (vs. CTRL). All data are the mean ± SD; *n* = 3, 4, and 5 for CTRL, GF, and UPy-GF, respectively. *MI* myocardial infarction

Histological analyses have been summarized in Table 1. As a reference, average cardiomyocyte diameter in the healthy pig heart was 18 ± 3 μm. Four weeks after the NOGA-guided injections, histological analysis revealed significant cardiomyocyte hypertrophy in the border zone of the CTRL hearts (Fig. 3a and Table 1). In contrast, both GF and UPy-GF treatments attenuated cardiomyocyte hypertrophy as well as increased the number of relatively small (<18 μm) cardiomyocytes compared to CTRL (18.47 ± 2.56 vs. 16.04 ± 1.85 vs. 21.20 ± 2.81 μm for GF, UPy-GF, and CTRL, respectively, *p* = 0.04; Fig. 3b, i). In line with previous pilot data [13], both the GF- and the UPy-GF-treated hearts showed a trend toward reduction in fibrosis, shown by picric Sirius red staining (Fig. 3c–h and Table 1), compared to the CTRL group (*p* = 0.27).

**Table 1:** Histological and immunohistological analysis at 1-month follow-up

	CTRL	IGF-1/HGF	UPy-IGF-1/HGF
Cardiac adverse remodeling			
CM Hypertrophy (μm)	21.2 ± 2.8	18.4 ± 2.6	16.0 ± 1.9*
Fibrosis (gray value per mm^2^)	40.7 ± 18.1	26.5 ± 13.7	25.8 ± 22.1
Proliferation			
Proliferation index (% Ki67^pos^ nuclei/total nuclei) (%)	0.3 ± 0.1	0.7 ± 0.3	1.1 ± 0.3*
Ki67^pos^ CM (border zone) (%)	0.03 ± 0.03	0.10 ± 0.03*	0.12 ± 0.03*
Cardiac stem cells			
c-kit^pos^ eCSCs (border zone) (%)	0.12 ± 0.1	0.14 ± 0.1	0.24 ± 0.1*
c-kit^pos^ eCSCs (infarct zone) (%)	0.13 ± 0.1	0.37 ± 0.1*	0.43 ± 0.1*
c-kit^pos^ Nkx2.5^pos^ eCSCs (border zone) (%)	25.2 ± 5.2	37.5 ± 5.9	45.5 ± 8.5*
c-kit^pos^ Nkx2.5^pos^ eCSCs (infarct zone) (%)	29.2 ± 10.0	33.7 ± 5.2	52.4 ± 14.8
c-kit^pos^ Ets-1^pos^ eCSCs (border zone) (%)	16.9 ± 3.6	20.2 ± 2.9	23.0 ± 4.0
c-kit^pos^ Ets-1^pos^ eCSCs (Infarct Zone) (%)	19.1 ± 5.1	24.2 ± 6.7	24.8 ± 5.3
Angiogenesis			
vWF^pos^ capillaries (border zone) (no. per 0.2 mm^2^)	6.3 ± 0.8	7.8 ± 0.9	8.7 ± 0.9*

Data are represented as the mean ± SD

*CTRL* controls, *IGF-1* insulin-like growth factor-1, *HGF* hepatocyte growth factor, *UPy* ureido-pyrimidinone, *CM* cardiomyocyte, *eCSC* endogenous cardiac stem/progenitor cell

*p < 0.05 (vs. CTRL); *p* < 0.05 (vs. IGF-1/HGF)

### Intramyocardial IGF-1/HGF Administration Leads to the Formation of New Cardiomyocytes

**Fig. 4:**
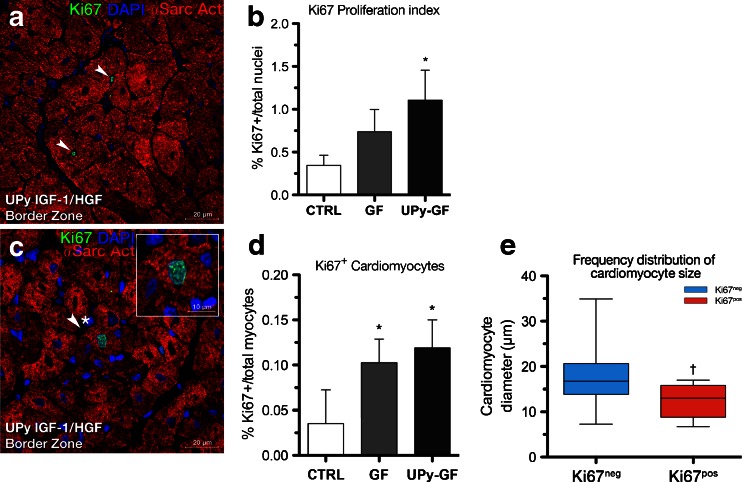
IGF-1/HGF administration leads to the formation of new cardiac myocytes. **a**, **b** Expression of cellular proliferation marker Ki67 (*green*) showed increased proliferation index of cells (*arrowheads*) in the UPy-GF-treated animals compared to CTRL. **c**, **d** Increased newly formed Ki67^pos^ (*green*) cardiomyocytes (*arrowheads*, *asterisk*; see *inset*) after GF treatment compared to CTRL in the peri-infarct/border zone. **e** Ki67^pos^ cardiac myocytes were smaller than the quiescent Ki67^neg^ cardiomyocyte fraction, indicative of their immature, newly formed nature. *p < 0.05 (vs. CTRL); ^†^ *p* < 0.05 (vs. Ki67^neg^ cardiac myocytes). All data are the mean ± SD; *n* = 3, 4, and 5 for CTRL, GF, and UPy-GF, respectively

Immunohistological analyses have been summarized in Table 1. Different myocardial cell types express growth factor receptors for IGF-1 and/or HGF. Thus, we sought to investigate the level of cell proliferation in the border zone of the chronic MI after GF treatment. Even 30 days after the injection procedure, an increased proliferation rate assessed by Ki67 expression was present within the GF-treated hearts, which was greater in the UPy-GF-treated hearts (Fig. 4a, b and Table 1). In particular, the border zone of the GF- and UPy-GF-treated animals harbored newly formed, small, immature Ki67^pos^ cardiomyocytes, which amounted to ∼1 every 1,000 cardiomyocytes (Fig. 4c). These small Ki67^pos^ cardiomyocytes accounted for >10 % of the total proliferating Ki67^pos^ cells in the GF-treated hearts, making their existence physiologically significant. Although Ki67^pos^ cardiomyocytes were also observed in the CTRL hearts, these were only witnessed in ∼1 every 3,000 cardiomyocytes (*p* = 0.016). To verify that these Ki67^pos^ cardiomyocytes were newly formed, we measured their size and compared this with Ki67^neg^ cardiomyocytes. Indeed, the Ki67^pos^ cardiomyocytes were on average smaller (12.52 ± 3.97 μm; Fig. 4e) compared to their Ki67^neg^ counterparts (17.48 ± 3.85, *p* = 0.0006; Fig. 4e), suggestive of a newly formed and immature cardiomyocyte subpopulation [6, 9].

### IGF-1/HGF Delivery Leads to the Formation of New Capillaries in the Infarct Borderzone

**Fig. 5:**
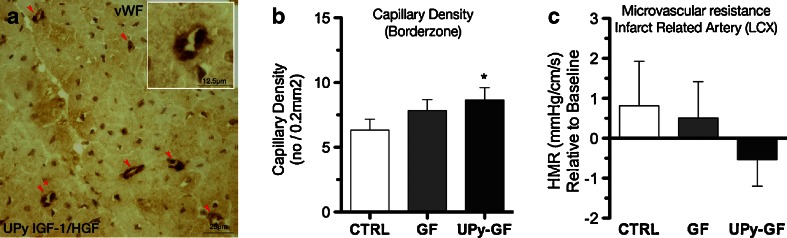
IGF-1/HGF leads to increased capillerization and reduces microvascular resistance. **a** Staining for von Willebrand factor (*vWF*) shows small capillary structures (*red arrowheads*, *asterisk*; see *inset*) in the border zone of the UPy-GF-treated heart. **b** Number of capillaries in the peri-infarct/border zone area. **c** Relative change, compared to baseline, in simultaneously measured intracoronary pressure and flow-derived hyperemic microvascular resistance (*HMR*). *p < 0.05 (vs. CTRL). All data are the mean ± SD; *n* = 3, 4, and 5 for CTRL, GF, and UPy-GF, respectively

The IGF-1/HGF treatment led to an increased number of capillaries in the infarct border zone, favoring the UPy-GF group (8.6 ± 0.9/0.2 vs. 7.8 ± 0.9/0.2 vs. 6.3 ± 0.8/0.2 mm^2^ for UPy-GF, GF, and CTRL, respectively, *p* = 0.022; Fig. 5a, b and Table 1). Consistent with the increased capillarization, the hyperemic microvascular resistance index (HMR; a simultaneously measured intracoronary pressure and flow velocity-derived parameter) was decreased in the infarct-related artery in the UPy-GF group compared to the HMR value measured just prior to the intramyocardial treatment delivery (*p* = 0.053; Fig. 5c).

### IGF-1/HGF Administration Leads to Expansionary Growth of the epCSC Compartment and Induces Cardiogenic Precursors

**Fig. 6:**
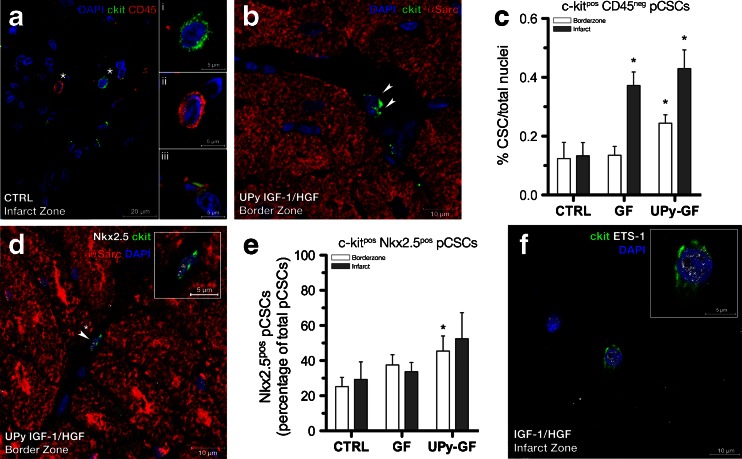
IGF-1/HGF treatment increases the epCSC compartment and drives their cardiac commitment in chronic MI. **a** The infarct area harbors various cell types, such as (*i*) c-kit^pos^ CD45^neg^ epCSCs, (*ii*) c-kit^neg^ CD45^pos^ cells, or (*iii*) c-kit^pos^ CD45^pos^ cells (including mast cells). **b** Endogenous epCSCs were a morphologically distinct subset of small cells showing perinuclear expression of c-kit (*green*, *arrowheads*) and negative for CD45. **c** Quantification of epCSCs in the peri-infarct/border and infarct zone. **d** A c-kit^pos^ (*green*) myogenic progenitor (*arrowhead*, *asterisk*; see *inset*) expressing the early cardiac transcription factor, Nkx2.5 (*white*). **e** Quantification of Nkx2.5^pos^ epCSCs in the peri-infarct/border and infarct zone. *p < 0.05 (vs. CTRL). All data are the mean ± SD; *n* = 3, 4, and 5 for CTRL, GF, and UPy-GF, respectively. **f** Some c-kit^pos^ epCSCs also expressed the transcription factor ETS-1 (*arrowhead*, *asterisk*; see *inset*)

To elucidate potential mechanisms governed by IGF-1/HGF stimulation that are responsible for the observed new cardiomyocyte and capillary formation, we determined the number and precursor state of the previously described c-kit^pos^ CD45^neg^ epCSCs [9]. We found increased c-kit^pos^ cells in the infarct and border zone with GF treatment; however, ∼73 % of all c-kit^pos^ cells also co-expressed CD45, identifying cardiac mast cells (iii in Fig. 6a and ESM Fig. 3) [9]. Furthermore, there was an infiltration of CD45^pos^ c-kit^neg^ cells into the infarct and border zone (ii in Fig. 6a). c-kit^pos^ CD45^neg^ epCSCs (i in Fig. 6a, b) had a relatively small cytoplasm-to-nuclei ratio, and in the infarct zone, the total number of epCSCs was increased fourfold by IGF-1/HGF delivery compared to CTRL hearts (0.37 ± 0.09 vs. 0.43 ± 0.14 vs. 0.12 ± 0.07 %, respectively, *p* = 0.004; Fig. 6c and Table 1). With regard to the border zone, the highest increase in c-kit^pos^ epCSC number was observed in the UPy-GF group (0.24 ± 0.06 %; Fig. 6c and Table 1) compared to GF or CTRL hearts (0.14 ± 0.06 vs. 0.12 ± 0.01 %, *p* = 0.03; Fig. 6c). Of those epCSCs, sustained IGF-1/HGF release induced a modest increase in the number of progenitor epCSCs (∼40 %) that co-expressed the early cardiac transcription factor Nkx2.5, indicative of their commitment toward the cardiomyogenic lineage (Fig. 6d, e and Table 1). Furthermore, another subset of epCSCs expressed the transcription factor Ets-1, indicative of their commitment to the endothelial lineage and the generation of capillaries (Fig. 6f and Table 1) [9].

## Discussion

In the present study, we sought to investigate the functional and histological/cellular effects of the intramyocardial administration of IGF-1/HGF in chronic MI in the pig. We show that improved delivery of IGF-1/HGF by a newly developed UPy hydrogel carrier holds potential as a novel treatment for chronic MI. Four weeks after delivery, UPy-IGF-1/HGF treatment led to a reduction in pathological cardiac remodeling, activated and increased the number of epCSCs, and led to the formation of new cardiomyocytes and capillaries. Importantly, the repair and regeneration of the damaged myocardial tissue was associated with a significant improvement in cardiac function.

### Heart Regeneration and eCSCs

To date, the presence of endogenous mechanisms for cardiomyocyte renewal in the postnatal heart remains a subject of intense debate [14]. Our findings presented here challenge the prevalent view that the adult mammalian heart, at best, can only increase its myocyte volume by means of a hypertrophic response of existing cardiac myocytes in the absence of new myocyte formation. Here, we show that the adult infarcted pig heart contains immature cardiac myocytes that are substantially smaller than normal, non-hypertrophied myocytes and do not reside in the quiescent G0 phase of the cell cycle, as would be expected given the hypothesis that the heart is a post-mitotic organ. Importantly, this regenerative potential of the adult heart could be effectively boosted by sustained release of the growth factors IGF-1 and HGF. These findings further ascertain the definitive presence of cardiomyocyte renewal in the adult mammalian heart as deducted from elaborate pulse-chase experiments published by various independent research groups [3, 7, 15–17].

Secondly, our present findings document that following IGF-1/HGF administration, the number of resident c-kit^pos^ epCSCs in the peri-infarcted area increased (Fig. 6) analogously to the increase in the presence of newly formed immature Ki67^pos^ cardiomyocytes (Fig. 4). Indeed, the majority of eCSCs in the peri-infarct region also co-expressed the nuclear transcription factors Nkx2.5 and Ets-1, indicative of their commitment toward the myogenic and vasculature lineage, respectively. However, as to what extent these newly formed cardiomyocytes reflect the differentiated progeny of eCSCs [7, 15] or whether they are the result of an endogenous regeneration mechanism that was indirectly mediated by paracrine actions [18, 19] could not be answered in this translational large animal model.

### Growth Factors to Stimulate Endogenous Cardiac Repair

Recently, essential growth factor/signaling pathways for cardiomyogenesis during the embryonic period have been summarized [20]. Various growth factors have been identified as potential candidates to guide postnatal stem progenitor cells toward a cardiomyogenic fate [8, 9, 21–23]. In a recent report by Chimenti and co-workers [19], the possibility was raised that eCSCs are not just mere consumers of growth factors but actively secrete a wide range of growth factors themselves, providing intricate networks of autocrine and paracrine feedback loops. We have previously documented that the effects of a single administration of IGF-1/HGF is still measurable 2 months after its application, suggesting the existence of a feedback loop triggered by the external stimuli that activates the production of growth and survival factors by the targeted cells, which explains the persistence and long duration of the regenerative myocardial response [9]. Since here we have observed effects on cell proliferation detectable 1 month after the delivery of a single dose of IGF-1/HGF, we speculate a similar autocrine/paracrine feedback loop that leads to sustained epCSC activation and proliferation and resultant cardiomyocyte formation, long after the primary stimulus has disappeared.

### Sustained Release of GF Using a Bioscaffold

Previous proof-of-concept experiments validating the UPy hydrogel showed that the hydrogel created a successful gradient of growth factors toward the infarcted area [13]. As a next step, the present study was undertaken to determine the therapeutic value provided by the sustained release of IGF-1/HGF using the UPy hydrogel carrier. This subsequent report advances initial findings by showing that IGF-1/HGF incorporated in the UPy hydrogel increased the effect of IGF-1/HGF therapy, but did not show statistical significance compared to equal concentrations of IGF-1/HGF dissolved in saline in both functional and histological endpoints (Fig. 2 and Table 1). However, when comparing the different growth factor-treated groups to the control-treated animals, only UPy-GF-treated animals showed improvement with statistical significance as opposed to the GF group in which significance was not reached for ESV and EF as well as several histological outcomes (i.e., CM hypertrophy, eCSC numbers in the MI border zone). Altogether, there is a highly consistent trend visible showing that the UPy-GF-treated animals outperformed the GF-treated animals on all levels of outcome measures (i.e., cardiomyocyte formation, number of c-kit^pos^ eCSCs, cardiac function).

### Clinical Perspective

By avoiding myocardial biopsies to extract eCSCs that need ex vivo upscaling to acquire clinically relevant numbers for subsequent delivery, one escapes from several drawbacks of cellular products as a novel treatment for ischemic heart disease [24, 25]. First and foremost, cellular therapy requires dedicated clinical centers that have both the expertise and high-cost resources for isolating, culturing, and handling stem cell products to pursue cardiac repair. Secondly, it relies on an available time span necessary for culturing stem/progenitor cells that is not present as in the case of *acute* myocardial infarction. Therefore, in situ activation of the eCSC compartment could bypass the aforementioned limitations of exogenous stem cell therapy. This holds true in particular for the chronic MI patients, in which aging and comorbidities also reduce the potency of the eCSC compartment. One particular aspect is the dramatic increase in cellular senescence of eCSCs to ∼70 % of all eCSCs in aged mice [26]. Work by Torella and colleagues [26] further showed that growth factors such as IGF-1 are capable of reversing this process in aged mice and restoring the function of aged senescent eCSCs.

Previous work on the therapeutic efficacy of IGF-1/HGF relied on transepicardial injections during open-chest surgery as the route of delivery [8, 10, 27]. In contrast, we used a percutaneous approach with the NOGA catheter system to acquire information on the infarct location and used the MYOSTAR catheter for targeted intramyocardial delivery in the peri-infarct/border zone of the chronic MI. As a consequence, the entire study protocol employed in this present work is clinically feasible and can be performed at a conventional catheterization laboratory. Work to address the use of UPy hydrogel synthesized under GMP conditions for human use is currently in progress.

### Limitations

Given the dynamicity in the temporal expression pattern of Ki67 in cycling cells, our histology, at best, provides a “snapshot” of cellular homeostasis in the post-MI heart at 1 month follow-up [28]. Therefore, we cannot draw inferences on the absolute number of newly formed cardiomyocytes in any of the treatment groups. Although we specifically characterized the contribution of tissue-specific c-kit^pos^ CD54^neg^ eCSCs, we cannot exclude that other stem/progenitor cell populations or other mechanisms of cardiomyogenesis contributed to new cardiac cell formation and, if so, to what extent. Furthermore, given the immature nature and low numbers of small, newly formed cardiomyocytes, the increase in cardiac function is most likely also caused by numerous other unknown factors, commonly designated as “paracrine effects” [29, 30]. The identification of these biological processes can provide further clues to improve growth factor-mediated cardiac repair and regeneration. Unraveling hereof is warranted in order to advance the cardioregenerative field to clinically relevant levels of myocardial regeneration.

Last, although experimental in vitro work on release by UPy hydrogel showed an ∼3-day sustained release of both IGF-1 and HGF, extrapolation toward the in vivo situation warrants certain caution. Since we did not choose to kill additional animals shortly after the GF injections, we cannot conclude whether the highest improvement in LV function seen in the UPy-GF group was actually caused by the sustained release of growth factors, or that the hydrogel was capable of retaining higher initial levels of growth factors compared to the saline solution. Despite careful placement of the intramyocardial injections, there is considerable backflow into the left ventricular cavity and/or venous drainage that could be potentially be minimized by the UPy hydrogel.

## Conclusion

In summary, four major conclusions can be deducted from this study: (1) targeted intramyocardial IGF-1/HGF injections attenuated pathologic cardiac remodeling and increased the formation of small, newly formed cardiomyocytes in the border zone of the infarct scar in the post-MI adult pig heart; (2) IGF-1/HGF admission gave rise to a robust increase of the c-kit^pos^ epCSC compartment of the heart and increased their commitment toward the cardiomyogenic and vasculature lineage; (3) intramyocardial IGF-1/HGF injections in the border zone of the infarct scar led to an improvement in cardiac systolic and diastolic function when compared to control-treated hearts; (4) the use of a smart hydrogel carrier that acts as a sustained-release platform increased the effectiveness of growth factor therapy as a treatment for chronic MI. Taken together, these results provide a rationale to further develop experimental work on growth factor therapy for myocardial repair and regeneration. Moreover, these findings identify the UPy hydrogel carrier system as a practical, affordable, and widely applicable therapeutic strategy designated to counteract the adverse remodeling and natural disease progression in the post-MI heart that would otherwise lead to congestive heart failure.

## Electronic supplementary material

Below is the link to the electronic supplementary material.

ESM 1 (DOCX 1069 kb)
