# Supplementary material for: Sustained Delivery of Insulin-Like Growth Factor-1/Hepatocyte Growth Factor Stimulates Endogenous Cardiac Repair in the Chronic Infarcted Pig Heart
Source: J Cardiovasc Transl Res. 2014 Jan 7;7(2):232–41. doi: 10.1007/s12265-013-9518-4 (PMC3935103; doi:10.1007/s12265-013-9518-4)
Supplement: Supplementary file 1 — (DOCX 1069 kb) [file 12265_2013_9518_MOESM1_ESM.docx]

**Online Appendix for the following Journal of Cardiovascular Translational Research article**

**TITLE:** Sustained Delivery of Insulin-Like Growth Factor-1/Hepatocyte Growth Factor Simulates Endogenous Cardiac Repair in the Chronic Infarcted Pig Heart.

**AUTHORS:** Stefan Koudstaal, MD, Maartje M.C. Bastings, PhD, Dries A.M. Feyen, MSc,

Cheryl D. Waring, PhD, Patricia Y.W. Dankers, PhD, Frebus J. van Slochteren MSc, Daniele Torella, MD, PhD, Joost P. Sluijter, PhD, Bernardo Nadal-Ginard, MD, PhD, Pieter A. Doevendans, MD, PhD, Georgina M. Ellison, PhD, Steven A.J. Chamuleau, MD, PhD

**Methods**

**UPy Hydrogel and Growth Factors**

The UPy-hydrogelators were synthesized by SyMO-Chem BV, Eindhoven, the Netherlands. ([1](#_ENREF_1)) For the preparation of the hydrogel, polymer solutions were dissolved at 10 wt% in phosphate buffered saline (PBS) by stirring at 70 °C for 2 hours and subsequently cooled to room temperature (RT). To liquefy the polymer solution, the pH was raised by adding 2 µL aliquots of a 0.1 M NaOH stock solution. Solutions were then UV-sterilized for at least 1-hour prior to use. Next, human recombinant HGF (Miltenyi Biotec) and human recombinant IGF-1 (Miltenyi Biotec) were mixed in by slow stirring for 10 minutes yielding a final concentration of 0.5 µg/mL of each growth factor.

**Animals and Study Design**

All in vivo experiments were conducted in accordance with the Guide for the Care and Use of Laboratory Animals prepared by the Institute of Laboratory Animal Resources. Experiments were approved by the Animal Experimentation Committee of the Medicine Faculty of the Utrecht University, the Netherlands. Eighteen female Dalland Landrace pigs (age ~6 months, weighing 68 ± 4 kg) were pretreated with clopidogrel 75 mg for 3 days and 500 mg acetylsalicylic acid one day before the operation. Prior to ischemia, the animals received 300 mg amiodarone and 5 mg metoprolol intravenously in 45 minutes to minimize the onset of cardiac arrhythmias. Myocardial infarction (MI) was induced by intracoronary balloon occlusion, under general anesthesia([2](#_ENREF_2)), of the proximal left circumflex artery for 75 minutes. Heparin was administered at 100 IU/kg body weight intravenously. Four weeks later, the animals underwent 3-dimensional electromechanical mapping (EMM) of the left ventricle for infarct and borderzone localization using the NOGA™ catheter system (Biosense Webster, Cordis, Johnson & Johnson, USA). Intramyocardial delivery was performed using the NOGA™ Myostar system (Biosense Webster, Cordis, Johnson & Johnson, USA), as previously described.([3](#_ENREF_3)) After randomized treatment allocation, ~10 injections of 0.2 mL were placed in the borderzone of the infarct with: 1) empty UPy-hydrogel (CTRL); 2) HGF/IGF-1 (0.5 µg ml^-1^/0.5 µg ml^-1^) (GF); 3) UPy-hydrogel loaded with HGF/IGF-1 (0.5 µg ml^-1^/0.5 µg ml^-1^) (UPy-GF). One month after the intramyocardial delivery, the animals underwent functional endpoint analysis and were sacrificed by exsanguination under general anesthesia. After excision of the heart, the left ventricle was cut into five slices from base to apex and incubated in 1% triphenyl-tetrazolium chloride dissolved in phosphate buffered saline (PBS) at 37°C for 15 minutes. Next, the slices were washed in PBS and photographed digitally (Sony Alfa 55). Infarct size was determined using ImageJ software (version 1.44g). The treatment allocation was kept blind to the investigator analyzing the infarct size.

**Intracoronary pressure and flow velocity assessment**

Intracoronary pressure and flow velocity were measured simultaneously by using the Combowire^®^ (Volcano Corporation, San Diego, USA) as previously described.([4](#_ENREF_4),[5](#_ENREF_5)) Pressure and flow velocity signals, combined with aortic pressure and ECG signals were recorded using the ComboMap^®^ system (Volcano Corporation, San Diego, USA). Intracoronary pressure and flow velocity were assessed prior to the infarction and four weeks after MI in the infarct related artery (LCX) and the reference artery (LAD). Nitroglycerin (200 mcg) was injected intracoronarily to prevent coronary spasms. Next, the Combowire was placed in the proximal section of the LCX and the LAD. Velocity and pressure signals were recorded during rest and maximal hyperemia. Hyperemia was induced by intracoronary bolus of 60 mcg adenosine. At least 3 representative measurements were performed per vessel.

Datasets were stored digitally and analyzed offline using AMC Study manager, a custom software package (written in Delphi vs. 6.0, Borland Software Corporation and Delphi vs. 2010, Embarcadero, CA, USA). CFVR was calculated as CFVR = pAPV / bAPV, where APV is average peak flow velocity in cm/s. The bAPV and pAPV were calculated as the mean of four beats at rest and the mean of three successive beats with the highest flow velocity respectively. HMR was calculated as HMR = P_d_ / pAPV, where both P_d_ and pAVP were derived from the mean of three beats at hyperemia.([6](#_ENREF_6)) The treatment allocation was kept blind to the investigator who performed the analysis.

**Echocardiography**

The echocardiographic examination was performed with the animals under general anesthesia, lying in the right lateral position. Ultrasound data was acquired using a Philips iE33 scanner (Philips Healthcare, Eindhoven, the Netherlands) with a S5-1 phased array transducer (1-5 MHz) for two-dimensional datasets and the X3 transducer for three-dimensional datasets. Standard parasternal long and short axis views were obtained. Left ventricular dimensions and wall thickness were measured in accordance with the standards of the American Society of Echocardiography.([7](#_ENREF_7)) Fractional area shortening was determined on the parasternal short axis view at papillary muscle level and expressed as a percentage of the LV internal area shortening in systole compared to the diastolic LV internal area. During the open-chest procedure at follow up, apical views (four-, two-, three-chamber) were obtained epicardially. Three-dimensional LV datasets were acquired under breath hold over six consecutive heartbeats to assess LV volumes and ejection fraction. Pulsed wave Doppler imaging was used to assess transmitral flow patterns. Tissue Doppler Imaging from the four-chamber view was used to determine the mitral annular motion at the basal part of the septum and lateral wall. Analysis included the average of three peak diastolic velocities (E’) for both annular sites combined. LV filling index was defined as the ratio of transmitral flow velocity to annular peak diastolic velocity (E/E’). The treatment allocation was kept blind to the investigator who performed the analysis.

**Pressure Volume loop analysis**

Pressure-volume (PV) loops were assessed using a 7-F conductance catheter. Briefly, LV pressure and volumes were measured and stored using a Leycom CFL-512 (CD-Leycom, Zoetermeer, the Netherlands). After correct placement in the LV, checked by the individual segmental conductance signals, the conductance signals were calibrated by cardiac output estimated by thermodilution of 5mL NaCl 0.9% at room temperature infused by Swan Ganz catheter. All data were collected while mechanical ventilation was paused. Data analysis and calculations were performed off-line using custom-made software (CD Leycom, Zoetermeer, the Netherlands), as described previously.([2](#_ENREF_2)) Systolic LV function was measured as LV ejection fraction, dP/dT^+^-peak, stroke work and preload recruitable stroke work. Diastolic function was assessed by dP/dT^-^ analysis. The treatment allocation was kept blind to the investigator performing the analysis.

**Immunohistochemistry**

Tissue sections were sampled from the infarct zone (visible as white area by TTC staining), border zone (viable myocardium directly adjacent to infarct zone) or the septal wall that served as remote area. Following fixation in 4% formalin, tissue samples were embedded in paraffin. Next, 7 µm sections were prepared on a TP1020 microtome (Leica). Hematoxylin and eosin staining was used for measurement of cardiac hypertrophy. The cardiomyocyte diameter of across the nucleus was measured in the borderzone of the infarct area on a light microscope (Nikon E1000M) using Lucia G software. A total of 100 cardiomyocytes/animal was measured. The mean value per animal was used in the subsequent analysis. Next, sections were incubated in 0.1% Fast Blue RR in magnesium borate buffer at pH 9 (Sigma) for 30 minutes. Following wash steps with distilled water, the slides were incubated in 0.1% Sirius red (BDH) in picric acid (Sigma) for 8 minutes at room temperature. After washing, the sections were dehydrated, cleared and mounted. The quantification was performed on 10 fields per animal under normal light and UV polarized light on a microscope (Olympos DP71) at 10x magnification and automatically quantified for the percentage of collagen per field using ImageJ software for Macintosh (version 1.44g). To quantify newly formed cardiomyocytes, tissue sections were stained for Ki67 (Vector), cardiomyocytes were identified by α-sarcomeric actin (Sigma) and nuclei stained by DAPI to identify cycling cells and cycling cardiomyocytes. In total 20 random fields of the peri-infarct borderzone were counted at 40x for cycling cells and cycling cardiomyocytes. To identify c-kit^pos^ CSCs, numbers of positive for c-kit (DAKO) and CD45 (Santa Cruz) was counted. DAPI was used to identify nuclei. Tissue sections were also stained with c-kit and Nkx2.5 (R&D) and ETS-1 (Santa Cruz) to identify lineage commitment. For the borderzone and infarct area, 5 random fields/area at 40x was counted. Capillary density was assessed by von Willebrand factor (vWF, Millipore) and counterstained with hematoxylin to identify nuclei. Vessels with a circumference spanning 1-3 endothelial cells were counted as capillaries.([8](#_ENREF_8)) In total, 10 fields/section at 40x were counted per animal in the borderzone. The amount of capillaries was expressed per 0.2mm^2^. Fluorescence images were visualized and acquired with confocal microscopy (Zeiss LSM710, LSM software). All analysis was performed by investigators who were blind to the group assignment.

**Statistics**

Continuous variables were presented as mean±SD unless stated otherwise and compared by one-way ANOVA. When a significant difference was found, post-hoc analysis was performed with Bonferroni’s test. When the assumption of the homogeneity of variance between groups was violated, nonparametric statistical analysis based on Kruskal-Wallis was performed. The assumption of a normal distribution was checked by QQ plots and the Kolmogorov-Smirnov test. To test whether the follow-up values differed from baseline values in each group, a paired T-test was used. All tests were performed using SPSS Statistics 17.0. Probability values of p <0.05 were considered statistically significant.

**References**

1. Dankers PYW, Hermans TM, Baughman TW, et al. Hierarchical formation of supramolecular transient networks in water: a modular injectable delivery system. Advanced Materials 2012;24:2703-9.

2. Timmers L, Henriques JPS, de Kleijn DPV, et al. Exenatide reduces infarct size and improves cardiac function in a porcine model of ischemia and reperfusion injury. J Am Coll Cardiol 2009;53:501-10.

3. van der Spoel TI, Vrijsen KR, Koudstaal S, et al. Transendocardial cell injection is not superior to intracoronary infusion in a porcine model of ischaemic cardiomyopathy: a study on delivery efficiency. J Cell Mol Med 2012;16:2768-76.

4. Chamuleau SAJ, Tio RA, de Cock CC, et al. Prognostic value of coronary blood flow velocity and myocardial perfusion in intermediate coronary narrowings and multivessel disease. J Am Coll Cardiol 2002;39:852-8.

5. Meuwissen M. Siebes M, Chamuleau SAJ, et al. Hyperemic Stenosis Resistance Index for Evaluation of Functional Coronary Lesion Severity. Circulation 2002;106:441-446.

6. Meuwissen M, Chamuleau SAJ, Siebes M, et al. Role of variability in microvascular resistance on fractional flow reserve and coronary blood flow velocity reserve in intermediate coronary lesions. Circulation 2001;103(2):184-7.

7. Lang RM, Bierig M, Devereux RB, et al. Recommendations for chamber quantification: a report from the American Society of Echocardiography's Guidelines and Standards Committee and the Chamber Quantification Writing Group, developed in conjunction with the European Association of Echocardiography, a branch of the European Society of Cardiology. J Am Soc Echocardiogr 2005;18:1440-63.

8. Ellison GM, Torella D, Dellegrottaglie S, et al. Endogenous Cardiac Stem Cell Activation by Insulin-Like Growth Factor-1/Hepatocyte Growth Factor Intracoronary Injection Fosters Survival and Regeneration of the Infarcted Pig Heart. J Am Coll Cardiol 2011;58:977-86

**Supplementary Information**

**
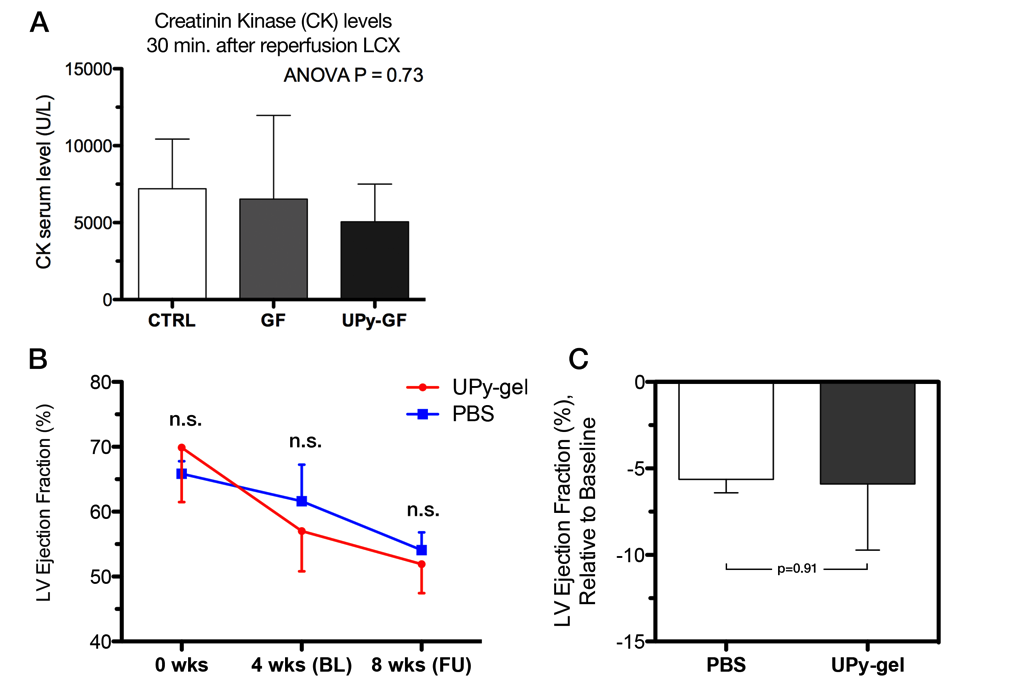
**

**Supplementary Figure 1. Myocardial Damage following MI and validation of empty UP-gel as negative control**

**(A)** Circulating levels of creatinin kinase (CK) 30 minutes after reperfusion of the coronary occlusion of the LCX shows no difference in the extent of myocardial damage between groups prior to the intervention four weeks later. **(B,C)** Comparison of empty UPy hydrogel injections against injections of phosphate buffered saline (PBS) shows no difference in LVEF at four weeks follow up.

**
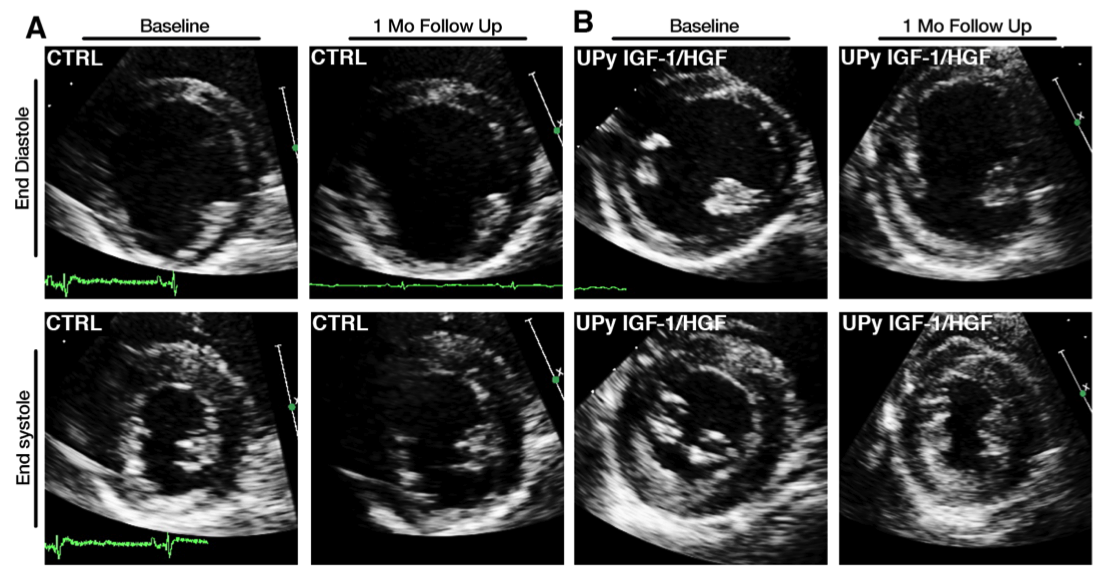
**

**Supplementary Figure 2. Echocardiographic data**

Representative 2D b-mode echocardiographic images of the short-axis view at the papillary muscle level showing the fractional area shortening for **(A)** CTRL and **(B)** UP-GF treated animals, at baseline and 1 month after intramyocardial delivery.

^
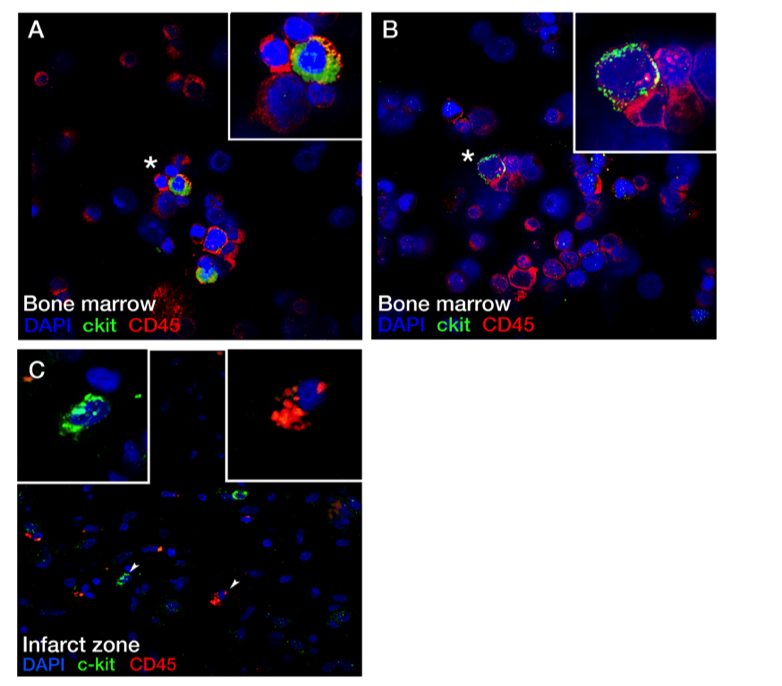
^

**Supplementary Figure 3. c-kit^pos^ CD45^neg^ eCSCs in the Porcine Heart**

**
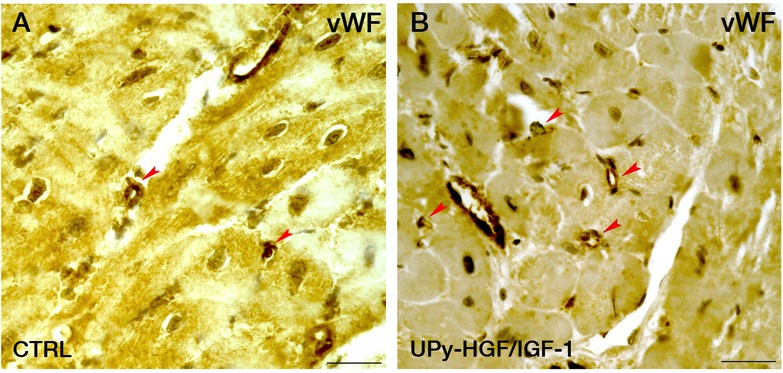
(A,B)** Representative confocal images of cytospin prepared porcine bone marrow cells co-expressing c-kit (green) and CD45 (red) or CD45 alone. **(C)** In the heart, c-kit^pos^ CD45^neg^ stem cells were observed, in addition to c-kit^pos^ CD45^pos^ and c-kit^neg^ CD45^pos^ cells (asterisks indicate inset).

**Supplementary Figure 4. HGF/IGF-1 Mediated Angiogenesis**

**(A,B)** vWF staining of the borderzone in CTRL (A) and UPy-HGF/IGF-1(B) treated animals show capillaries (red arrowheads) as defined by 1 or 2 endothelial cells spanning the vWF positive vessel circumference (shown by DAB staining in brown). Scale bar represents 25 µm.
